# Down-regulation of miR-181c in imatinib-resistant chronic myeloid leukemia

**DOI:** 10.1186/1755-8166-6-27

**Published:** 2013-07-16

**Authors:** Neda Mosakhani, Satu Mustjoki, Sakari Knuutila

**Affiliations:** 1 Department of Pathology, Haartman Institute, and HUSLAB, University of Helsinki and Helsinki University Central Hospital, Helsinki, Finland; 2 Hematology Research Unit Helsinki, Department of Medicine, Division of Hematology, University of Helsinki and Helsinki University Central Hospital, Helsinki, Finland

**Keywords:** miRNA, CML, Imatinib response

## Abstract

The association of microRNA alterations with progression and treatment outcome has been revealed in different types of cancers. To find miRNAs involved in imatinib response we performed miRNA microarray followed by RT-qPCR verification of 9 available diagnostic bone marrow core biopsies from 9 CML patients including 4 imatinib-resistant and 5 imatinib-responder patients. Only one differentially expressed miRNA, *miR-181c*, was found when the imatinib-resistant group was compared with imatinib-responders. Significant down-regulation of *miR-181c* in imatinib-resistant versus imatinib-responders was confirmed by qRT-PCR. Some *miR-181c* target genes such as *PBX3*, *HSP90B1, NMT2* and *RAD21* have been associated with drug response*.*

## 

Chronic myeloid leukemia (CML) is characterized by unregulated proliferation of myeloid cells in the bone marrow that carry the *BCR-ABL* fusion gene. In most of the patients, the ABL tyrosine kinase of the fusion protein is effectively inhibited by the tyrosine kinase inhibitors (TKIs), but some patients are resistant to TKI therapy. Whereas the *BCR-ABL* fusion drives the initial chronic phase of the disease, the progression of CML involves additional genomic changes which make leukemia cells resistant to TKI therapy and independent of *BCR-ABL*. Recently, in a variety of cancers the role of microRNAs (miRNA) in disease progression has been addressed [1]. MiRNAs are potential regulators of drug efficacy, because they target many important drug-related genes [2].

To understand which miRNAs are associated with the TKI therapy response, we performed miRNA microarray in 9 bone marrow core biopsies derived from 9 CML patients at diagnosis including 5 imatinib-responder and 4 imatinib-resistant patients. The ABL mutations were not tested at the time of diagnosis, and during the therapy they were only tested for resistant patients. Three of four patients developed mutations later during the treatment, but this occurred 6–10 years after the diagnosis. The study was conducted in accordance with the principles of the Helsinki Declaration and was approved by the Helsinki University Central Hospital Ethics Committee. Written informed consent was obtained from each patient. For clinical information see Table 1.

**Table 1:** Patient characteristics

**Patient no.**	**Year of dg**	**Gender**	**Age at dg**	**Sokal score**	**Treatment**	**Treatment response**
1.	1996	F	45	7.44	HU, 11/96 IFN, 6/2002 IM, 5/2005 DAS	No CgR
2.	1998	F	56	1.37	HU, 3/99 IFN, 04/2002 IM, 08/2006 DAS	Min CgR
3.	2010	M	68	1.61	HU, 3/2010 IM, 12/2010 NIL, 6/2011 DAS	Min CgR
4.	2008	M	58	1.39	HU, 7/2008 IM, 6/2009 DAS, 3/2010 alloHSCT	No CgR
5.	2005	M	48	0.93	HU, 11/2005 IM, 2/2006 IM + IFN	CMR
6.	2005	M	58	0.77	11/2005 IM	CMR
7.	2004	M	55	0.69	HU, 10/2004 IM, 3/2005 IM + IFN	CMR
8.	2006	F	53	0.68	HU, 1/2007 IM	CMR
9.	2006	M	52	0.85	HU, 1/2007 IM	MMR

In all non-responder patients (patients 1–4) the treatment response to imatinib was failure based on the European Leukemia Net (ELN) criteria [3] (no or minimal cytogenetic response to imatinib within 12 months after the start of therapy). These patients were subsequently treated with other TKIs (dasatinib/nilotinib), but no cytogenetic responses were achieved with 2nd generation TKIs either, confirming these patients to be non-responders to multiple TKIs. Patients in the responder group (patients 5–9) fulfilled the optimal response to imatinib based on ELN criteria. Abbreviations: *no* number, *dg* diagnosis, *HU* hydroxyurea, *IFN* interferon, *IM* imatinib, *DAS* dasatinib, *NIL* nilotinib, *alloHSCT* allogeneic hematopoietic stem cell transplantation, *CgR* cytogenetic response, *min* minimal, *CMR* complete molecular response, *MMR* major molecular response.

From core biopsies, total RNA, including miRNA, was isolated with the miRNeasy FFPE Mini Kit (Qiagen, Valencia, CA, USA). To check the quality of total RNA we used the RNA 6000 chip and for miRNA the small RNA chip (Agilent Technologies, Santa Clara, CA, USA) Agilent’s Bioanalyzer. An miRNA microarray system (V3) (containing 866 human and 89 human viral miRNAs) (Agilent) was used for miRNA profiling according to Agilent’s protocol. Based on our previous study, the core biopsy samples are a reliable source for miRNA profiling [4].

The raw data were analyzed with GeneSpring Software v.11.5.0. The data were preprocessed by taking log2 and normalized by the 75th percentile method. The T-test was applied to find the most significant differentially expressed miRNAs (*P*<0.05 and False discovery rate or q < 0.05).

Despite the small sample size used in our study—which indicates the rarity of resistant patients—we found one miRNA, *miR-181c,* which is differentially expressed between imatinib-resistant and imatinib-responder patients (*P*=1.41E-6, q=6.1E-4). *MiR-181c* was validated by quantitative RT-PCR (qRT-PCR) by the use of the SYBR Green miScript PCR system (Qiagen) on the Light-cycler, software v.3.5 (Roche Applied Science, Mannheim, Germany). The primer sequence for *miR-181c* was purchased from Qiagen and the primer was 5' AACAUUCAACCUGUCGGUGAGU. The snRNA U6 gene (Qiagen) served as the normalization control, and relative quantification for each miRNA was calculated using the 2^−ΔΔCt^. Significant down-regulation of *miR-181c* (p=0.04) in imatinib-resistant vs. imatinib-responder patients was confirmed by qRT-PCR (Figure 1 and Additional file 1: Figure S1).

**Figure 1:**
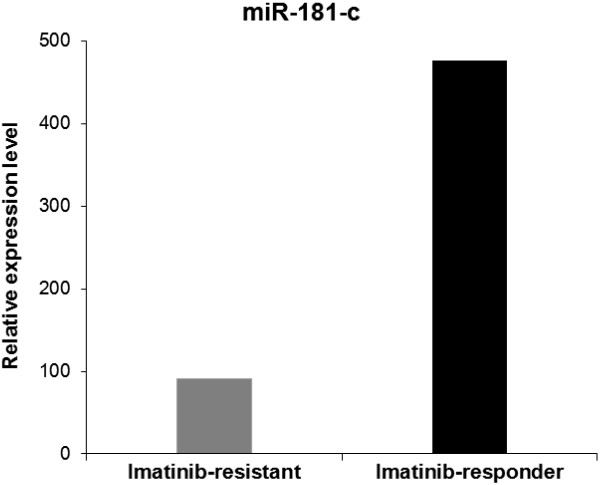
**Average of relative expression level of *miR-181c *expression.***miR-181c* is down-regulated in imatinib-resistant compared to imatinib-responder samples.

In normal conditions, *miR-181c* is expressed in the thymus, primary lymphoid organs, brain, lungs, bone marrow, and spleen [5]. *MiR-181* has also been associated with the differentiation of both hematopoietic B cells [5] and T cells [6], and myoblasts [7]. *MiR-181c* is involved in a tumor-suppression pathway [8] and likely in regulation of the Rb pathway which mediates cell-growth arrest [9]. Its inhibitory effect on cell growth and increasing apoptosis has been observed in glioma cells [8]. In accordance with our finding, the strong down-regulation of the *miR-181* family, including *miR-181c,* has been observed in Lyn-mediated imatinib-resistant CML cells [10]. Similarly, AML patients with intermediate- or poor-risk subtypes have been reported to have lower *miR-181* levels than do patients with favorable prognosis [11]. The decreased expression of this miRNA has also been found in Fanconi anemia patients, and been involved in the impaired growth of their hematopoietic progenitors [12]. However, in two recent studies involving CML patients, no association appeared between the *miR-181c* expression and imatinib therapy response [13,14]. Furthermore, in CML patients with blast chrisis, the *miR-181c* was not differentially expressed, but *miR-181a* and *miR-181b* were upregulated [15].

To study which genes are targeted by *miR-181c,* we used Chipster software v.1.4. To reduce false positivity, target genes needed to be predicted by at least five of six algorithms, including TargetScan, miRanda, Sanger miRBase, mirTarget2, Tarbase, and PICTAR (Additional file 2: Table S1). Some of the *miR-181c* target genes are associated with prognosis and drug response, ones such as *PBX3*, *HSP90B1, NMT2,* and *RAD21*. For example, AML patients with intermediate- to poor-prognosis subtypes, who had lower expression of *miR-181*, conversely showed an increased level of *PBX3*[16]. The up-regulation of *NMT2* and *RAD21* contributes to chemoresistance in osteosarcoma cell lines and also in breast cancers [17,18]. Similarly, high expression of HSP90B1 is associated in breast cancer with distant metastasis and with decreased overall and disease-free survival [19]. Moreover, high HSP90 expression predicts worse overall survival in patients with acute lymphocytic leukemia [20].

In conclusion, *miR-181c* associated with imatinib resistance. Larger sample sizes and further independent studies, however, are warranted to assess the role of candidate miRNA and target genes in the molecular mechanisms underlying resistance in CML.

## Abbreviations

TKIs: tyrosine kinase inhibitors.

## Competing interests

The authors declare that they have no competing interests.

## Authors’ contributions

SK, as a senior researcher, designed the study and participated in writing the manuscript. NM performed the laboratory work and participated in writing. SM participated in designing the study and provided clinical data and preparing the manuscript. All authors read and approved the final manuscript.

## Supplementary Material

Additional file 1: Figure S1 Relative expression level of *miR-181c* in individual samples. Click here for file

Additional file 2: Table S1 Predicted target genes by at least five databases for *miR-181c.* Click here for file
