# Supplementary figures and images for: Down-regulation of miR-181c in imatinib-resistant chronic myeloid leukemia
Source: Mol Cytogenet. 2013 Jul 16;6:27. doi: 10.1186/1755-8166-6-27 (PMC3751646; doi:10.1186/1755-8166-6-27)

**Supplementary Figure 1**

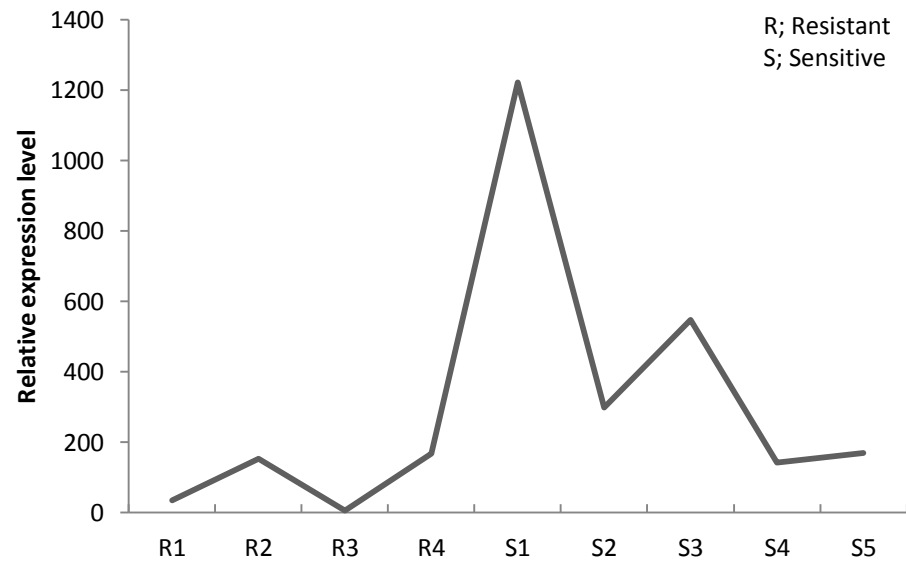

Supplement: Additional file 1: Figure S1 — Relative expression level of miR-181c in individual samples. [file 1755-8166-6-27-S1.pdf]
