# Supplementary material for: Down-regulation of miR-181c in imatinib-resistant chronic myeloid leukemia
Source: Mol Cytogenet. 2013 Jul 16;6:27. doi: 10.1186/1755-8166-6-27 (PMC3751646; doi:10.1186/1755-8166-6-27)
Supplement: Additional file 2: Table S1 — Predicted target genes by at least five databases for miR-181c. [file 1755-8166-6-27-S2.pdf]

Supplementary Table S1. Predicted target genes by at least five databases for *miR-181c*

ACSL1	LRRC8D
ADAMTS6	NMT2
ARSJ	NR6A1
BAI3	PAWR
BRD1	PBX3
CLIP1	PCDHA1
DOCK7	PHF20L1
EIF4A2	PRKCD
FAM160A2	RAD21
FAM179B	RAN
FKBP1A	RBBP7
FNDC3A	RLF
GABRA1	RNF145
HSP90B1	RNF34
ITGB8	TBPL1
KIAA0195	TCERG1
KIAA0528	TOM1L1
KLF6	TSC22D2
KLHL5	ZFAND6
KPNB1	PPIP5K2
